# Sources of Microplastic Generation in the Environment

**DOI:** 10.3390/ijerph20136202

**Published:** 2023-06-22

**Authors:** José Machado Moita Neto, Elaine Aparecida da Silva

**Affiliations:** 1 Universidade Federal do Delta do Parnaíba—UFDPar, Ministro Reis Velloso Campus, Parnaíba 64202-020, Brazil; jose.machado.moita.neto@gmail.com; 2 Department of Water Resources, Geotechnics and Environmental Sanitation, Universidade Federal do Piauí—UFPI, Ministro Petrônio Portella Campus, Teresina 64049-550, Brazil

## 1. Relevance of Microplastics as an Environmental Issue

The number of scientific papers on microplastics in the environment has grown exponentially. A search in one of the world’s largest databases (Science Direct) registered more than 5000 research articles by May 2023 (5526 on 21 May 2023: microplastics in title, abstract, and keywords; research articles as article type; and Environmental Sciences as the subject area).

**Figure 1:**
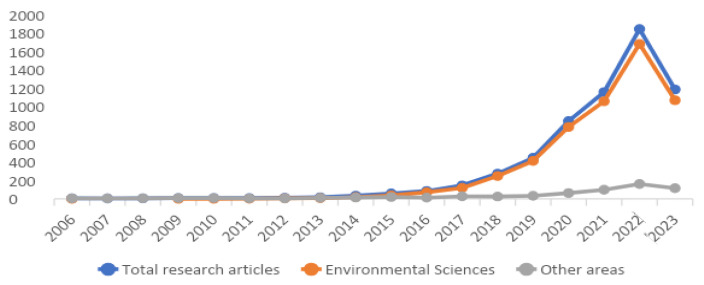
Annual distribution of published articles on microplastics, according to the Science Direct database [1].

As shown in Figure 1, when comparing the production of scientific publication in the area of Environmental Sciences and other areas (Earth and Planetary Sciences; Agricultural and Biological Sciences; Chemical Engineering; Materials Science; Engineering; Energy; Chemistry; Biochemistry, Genetics and Molecular Biology; and Pharmacology, Toxicology and Pharmaceutical Science), the year 2014 marks the predominance of the theme microplastics in the area of Environmental Sciences, and this is consolidated in the following years to date.

An understanding of the political relevance of this topic can be found in the European Commission’s effort to contemplate it in various initiatives that started in 2019 with the Publication of the European Green Deal [2].

## 2. General Information on Polymeric Materials

There is a multitude of types of polymeric materials in nature, and many more can be achieved synthetically. Synthetic polymers can be obtained with a wide variety of chemical and physical properties. In addition, the use of additives and blends or the possibility of forming composites opens up a range of possibilities for plastics to become a suitable and viable alternative to almost any type of material or application. This explains their omnipresence in all environments.

The generic denomination of plastics to products with polymeric materials that are obtained synthetically in their composition has become popular, but such classification is technically inadequate. The differences between polyethylene (PE), polyethylene terephthalate (PET) and engineering plastics, for example, are not only in their chemical and physical properties but also in the way of reusing and recycling these products for a post-use stage.

There is, however, a more pressing concern than esthetics when one considers the real possibility of plastics fragmenting into micrometric sizes in the environment. The simple act of sweeping a surface leads to the wear and tear of the material that makes up the part of the broom that comes into contact with the surface. Straw and synthetic polymer fiber have different reactivities in the environment; by comparing the two, it can be seen that straw is biodegradable and polymer fibers are not. Both materials will end up in the soil or carried by the wind and perhaps precipitate into a body of water. This uncontrollable path is longer lasting for synthetic materials that are inert to environmental conditions.

Despite the differences between polymeric materials, a common property is their great chemical inertness, that is, the difficulty in transforming or breaking down polymeric materials into chemically smaller units under environmental conditions. The degradation of plastics into physically smaller units (down to the dimensions of microplastics or nanoplastics) happens for all polymeric materials. There are adverse environmental conditions, to which these materials’ polymer chains are resistant, that are capable of damaging their structural stability via degradation or via the loss of smaller (non-polymeric) molecules that constitute the various additives of plastic materials.

The drying out of plastic materials by the loss of small molecules that are plasticizer additives is one of the most common and well-known phenomena. From electric cables to plastic chairs, plastic materials lose their condition of use under continuous exposure to weather conditions, thus becoming fragile from the point of view of their mechanical properties. Therefore, in general, the disposal of a plastic product happens due to mechanical reasons (physical) or biological contamination, and not for chemical reasons. For comparison, a material like steel undergoes corrosion (chemical attack) long before its mechanical properties are compromised for its intended purpose.

## 3. Differences between Nanoplastics and Microplastics

The comparison between microplastics and nanoplastics must not focus only on their chemical structure but should take into account two crucial factors: (a) the size and (b) the surface properties of their particles. By volume exclusion, nano-sized particles can penetrate into places where micrometric particles are intercepted. In addition, nanoscale particles acquire surface properties that can transform them in relation to their electrical charge and the transportation of other materials, including viruses and bacteria, on their surface.

The fragmentation of a micrometric material into a nanometric material expands the surface of contact by 1000 times in a medium, enabling the appearance of various phenomena that are typical of the colloidal dimension of particles, including the modification of charge and surface energy. Nanoplastics, for the reasons mentioned above, can affect living beings more seriously than microplastics.

## 4. Inventory of Potential Microplastic Sources

The inventory of microplastic sources can be managed through a description of the processes and mechanisms to which plastic materials are subjected and which can lead to the incorporation of microplastics into the environment. Obviously, not all of these sources have the same weight in terms of the amount of existing microplastics in them. A qualitative approach to these sources, without a corresponding quantitative approach, is too limited to attain an assertive answer that can achieve effective regulation and prevent the mismanagement of plastic waste with the potential to become microplastics or nanoplastics.

Nevertheless, the best way to approach this issue is to classify and present ways that are known to potentially generate microplastics, considering current scientific knowledge and technologies of these materials and other similar processes. In this sense, the inventory of microplastic sources presented in this paper is primarily conceptual and qualitative. The main processes and mechanisms of microplastic generation are presented below.

### 4.1. Air Flows

The connection that we can make between rock formations that have been shaped by wind over time, which have become tourist attractions in various parts of the world, and the subject of microplastics is almost evident. In the same way that the wind’s action in continuously corroding rock is only noticeable after a long period of time, all structures containing polymeric materials will also suffer this erosion, and the fragments of these materials will be blown away by the wind itself.

A car, for example, has plastic materials exposed to the external environment, which are subjected to wind action amplified by the vehicle’s own speed in motion.

### 4.2. Water Flows

The water that flows over any surface has the ability to drag and sometimes tear off what is in its path. Erosion caused by water is well documented in rocks and soil. The mechanical action of simple water runoff on a polymeric surface exerts a shearing force on the material that can lead to fragmentation. In addition, systems designed to allow water to have a main or complementary mechanical action through the simple transport by water flow are also subjected to wear.

As an example, in a domestic washing machine, both the parts of the machine and what is being washed are subjected to the mechanical action of water. This kind of wear also happens to other materials and in other situations, such as the wear of concrete in a gutter by the passage of rainwater.

### 4.3. Chemical Oxidation

Due to their great inertia (low chemical reactivity), synthetic plastics do not suffer chemical wear in the environment in the same way that steel, aluminum, copper and other metals and alloys do. Polymeric materials present chemical stability to environmental conditions similar to ceramic materials, although they have other chemical behavior. This chemical inertness of polymeric materials is reflected by their presence in the environment during and after use. However, such materials can easily be attacked or modified in laboratories or industrial plants for this purpose. When exposed to environmental conditions, only surface oxidation of polymeric materials is possible, which is facilitated by the action of ultraviolet rays that exist in small quantities in the solar spectrum.

Oxo-biodegradable bags, depending on the amount of additives placed in the polymeric material used, will fragment to micrometric dimensions and, in some cases, reach nanometric dimensions. However, as they are not attacked by microorganisms and because the chemical structure of plastics remains the same in nanoplastics, the esthetic problem of the visibility of these bags disappears, while the real problem of plastics in the environment becomes more serious. In this way, the wear experienced on these materials is not exactly chemical wear but physical wear that potentializes them to become a source of microplastics in the environment.

### 4.4. Mechanical Fragmentation

Due to the required use of products containing plastic materials or via the improper exposure of plastic products to solar radiation, there will be breakage of some chemical bonds and superficial oxidation of these materials. This embrittlement accumulates over time and compromises the mechanical properties of plastic materials. Throughout the process, there may be physical fragmentation of such materials, and these fragments will be carried by the wind as microplastics.

The proper use of a plastic material may require the need for some mechanical action on its part, such as drilling or grinding. While such action does not produce microplastics immediately, it greatly increases the reactive surface area of the material, which can subsequently generate microplastics in the environment. As an example, when you see a bald tire, you can imagine that its old treads have been fragmented by friction with sidewalks and the fragments are carried away by the wind, constituting another source of microplastics.

Paradoxical as it may seem, many plastic materials require additives called plasticizers. These are small molecules (unlike plastics!) that exert an internal lubrication between polymer chains, thereby conferring better properties for use or for the processing of materials. The initial, nearly homogeneous distribution of plasticizers within plastics changes over time due to the slow migration of these small molecules to the surface. This diffusion process is natural and controlled by well-known thermodynamic and kinetic factors. The result of this diffusion is the loss of material properties with apparent drying out, which renders the material brittle.

## 5. Environmental Education and Management

The economic management of a company is of direct interest to the company itself and to the government, which regulates the sector and equalizes competition among market agents. In addition, every enterprise must pay taxes to enable direct or indirect government action on behalf of the society as a whole. Environmental management is also in the interest of the society because any failure in a company’s management or in the government’s role will, at the very least, trespass on the diffuse rights of citizens to have a healthy environment for themselves and future generations.

The proper management of the plastics industry, by obligation, must include reverse logistics because the best destination for plastic waste is to enter another industrial plant for reprocessing, incineration or energy production. The compulsory nature of reverse logistics can increase costs, which are certainly lower than the environmental costs that our society pays today for the accelerated production of microplastics.

Current selective waste collection alternatives for plastics are insufficient because these materials are processed in the same way, such as by treating them as if they are a simple mixture of polyethylene and polypropylene. Only reverse logistics, which enables the return of plastics to the company that introduced them into the market, can optimize the reuse of these materials after they have been used.

When a theme becomes important enough to have political, economic and environmental repercussions, it is also placed in the public space for discussion. It becomes an object of dispute with various vested interests at stake, and there is a race to schedule social media posts in an attempt to impose one’s own vision on the society.

The war waged against plastic straws and plastic bags is an example of environmental problems that has been misplaced, taking away the main focus of our society on the waste management of plastics. The villainizing of these products in a simplistic view that ignores processes and substitutes, while disregarding environmental management and education, shows how much our society is being held hostage to its technical and scientific ignorance about plastic materials.

Environmental education on the subject of microplastics involves knowledge of their production in the use and post-use of polymeric materials, possibilities of implementing reverse logistics, knowledge of the performance of other materials (advantages and disadvantages) and, above all, understanding that the complexity of the issue does not allow simplistic solutions. In addition, environmental education should develop critical thinking to evaluate the multitude of debates and discussions on the subject that nowadays dominate social media.

One of the goals of the European initiative on microplastics is to “reduce the unintentional release of microplastics in the environment” [2]. There is an understatement in this because “intent” has a subjective character and can be extended to all imaginable sets of sources for which sufficient technical knowledge is already available.

Knowledge of Materials Science allows one to identify the quantity of primary and secondary sources of microplastics in the environment. Tests that mimic environmental conditions and accelerate them can simulate adverse conditions that lead to the fragmentation of polymeric materials. This means that the goal of reducing the release of microplastics into the environment should be a regulatory one for the industry and an educational one for society.

## 6. Final Remarks

Isolating microplastics among different materials and presenting microplastics as a problem to be solved does not adequately address a common problem that any material wears out with use. For example, in addition to reducing friction between two metallic parts, the function of a lubricating oil is to receive the metallic fragments of this wear during use. This happens not only in use but also in the preparation of any material. Cutting, carving, sawing, sanding, polishing, grinding and many other processes can be understood as generators of fragments that will go into the environment, regardless of whether the application is on wood, ceramic or metal.

The number of scientific studies that point out problems posed by plastics to human health and the environment cannot be underestimated. However, the situation is much more complex than one might think, and the solution requires an inventory of all possible sources, the determination and quantification of plastics in the environment, and the development of international indicators for monitoring and subsequent standardization of the most serious cases. Environmental education about plastics and their proper management is also a long-term action that can and must be taken. What needs to be prevented are the anti-scientific and anti-technological attitudes against a class of materials with adjustable properties, which can be subjected to regulated management and have better performance than all other materials that compete with them in several applications.
